# Muscle Arnt/Hif1β Is Dispensable in Myofiber Type Determination, Vascularization and Insulin Sensitivity

**DOI:** 10.1371/journal.pone.0168457

**Published:** 2016-12-22

**Authors:** Pierre-Marie Badin, Danesh H. Sopariwala, Sabina Lorca, Vihang A. Narkar

**Affiliations:** 1 Metabolic and Degenerative Diseases, Brown Foundation Institute of Molecular Medicine, McGovern Medical School, UTHealth, Houston, TX, United States of America; 2 Integrative Biology and Pharmacology, McGovern Medical School, UTHealth, Houston, TX, United States of America; 3 Graduate School of Biomedical Sciences, McGovern Medical School, UTHealth, Houston, TX, United States of America; Institut d'Investigacions Biomèdiques August Pi i Sunyer, SPAIN

## Abstract

Aryl Hydrocarbon Receptor Nuclear Translocator/ hypoxia-inducible factor 1 beta (ARNT/ HIF1β), a member of bHLH-PAS family of transcriptional factors, plays a critical role in metabolic homeostasis, insulin resistance and glucose intolerance. The contributions of ARNT in pancreas, liver and adipose tissue to energy balance through gene regulation have been described. Surprisingly, the impact of ARNT signaling in the skeletal muscles, one of the major organs involved in glucose disposal, has not been investigated, especially in type II diabetes. Here we report that ARNT is expressed in the skeletal muscles, particularly in the energy-efficient oxidative slow-twitch myofibers, which are characterized by increased oxidative capacity, mitochondrial content, vascular supply and insulin sensitivity. However, muscle-specific deletion of ARNT did not change myofiber type distribution, oxidative capacity, mitochondrial content, capillarity, or the expression of genes associated with these features. Consequently, the lack of ARNT in the skeletal muscle did not affect weight gain, lean/fat mass, insulin sensitivity and glucose tolerance in lean mice, nor did it impact insulin resistance and glucose intolerance in high fat diet-induced obesity. Therefore, skeletal muscle ARNT is dispensable for controlling muscle fiber type and metabolic regulation, as well as diet-induced weight control, insulin sensitivity and glucose tolerance.

## Introduction

The Aryl Hydrocarbon Receptor Nuclear Translocator (ARNT), also known as the hypoxia-inducible factor 1 beta (HIF1β), is a transcription factor belonging to the basic helix-loop-helix, Per/AHR/ARNT/SIM (bHLH-PAS) family [1]. The ARNT/HIF1β (henceforth referred to as ARNT) primarily contributes to gene regulation by forming transcriptional heterodimers with other members of the same family such as HIF (e.g. HIF1α and HIF2α), the aryl hydrocarbon receptor (AHR), Single-minded homolog (e.g. Sim 1 and Sim 2), Period (e.g. Per 1, Per 2, Per 3). Speculatively, ARNT interactions may not be limited to bHLH-PAS members, and ARNT could heterodimerize with other transcriptional regulators, expanding its scope in transcriptional regulation. Accordingly, ARNT could have a major physiological function, including the regulation of hypoxic response, brain development, xenobiotic response and circadian rhythm through respective interaction with HIF, AHR and SIM, and PER proteins. Additionally, ARNT has been shown to be involved in angiogenesis, cell survival and lipid homeostasis [2–5].

Recent studies have highlighted the critical role of ARNT in energy homeostasis in major endocrine organs, and consequently in the pathogenesis of type II diabetes. ARNT expression is dramatically down-regulated in pancreatic islets from humans with type II diabetes. Selective deletion of ARNT in beta cell of the pancreas leads to glucose intolerance, impaired insulin secretion and deregulated islet gene expression [6,7]. Other studies using conditional knockout mice have additionally reported the critical role of pancreatic ARNT in beta cell transplant, and glucose tolerance during pregnancy [8,9]. ARNT expression is also repressed in diabetic livers. Liver-specific deletion of ARNT increases hepatic gluconeogenesis, dyslipidemia, glucose intolerance and insulin resistance. In contrast to its role in pancreas and liver, deletion of ARNT in adipose tissue actually improves glucose tolerance and insulin sensitivity, potentially via its effect of hypoxic response, angiogenesis and vasculature in the adipose tissue [10,11].

Surprisingly, the contribution of skeletal muscle ARNT to metabolic homeostasis and development of insulin resistance has not been explored. The rationale for examining the contribution of muscle ARNT to myofiber specification, obesity, and glucose homeostasis emerges from several observations. As mentioned above, manipulation of ARNT expression in various organs relevant to energy homeostasis such as pancreas, liver and adipose tissues influenced glucose intolerance and insulin resistance [6,10–12]. Skeletal muscle accounts for approximately 30–50% of the body weight, and is a critical organ involved in glucose homeostasis, as it disposes approximately 80% of glucose during an hyper-insulinemic euglycemic clamp [13]. Type II diabetes is partly a result of lipid deregulation and development of insulin resistance, leading to impaired insulin-dependent glucose uptake in the skeletal muscle. Generally, the capacity of insulin-stimulated glucose disposal seems to directly correlate with oxidative slow-twitch myofiber proportion, functional mitochondrial content, and oxidative capacity in the skeletal muscle [14]. Both HIF1A and HIF2A, which utilize ARNT in the heterodimer transcriptional complex, influence skeletal muscle myofiber type and metabolic capacity. HIF1A controls glycolysis in the skeletal muscles, and muscle-specific deletion of HIF1A causes a compensatory increase in oxidative and fatty acid metabolism [15]. On the other hand, HIF2A encodes an oxidative slow-twitch muscle program in the skeletal muscle, and deletion of muscle HIF2A triggers a shift to more glycolytic fast-twitch muscles [16]. Thus, HIF1A and HIF2A collectively control both the glycolytic and oxidative arms of muscle metabolism, additionally impacting myofiber type specification. Signaling pathways regulating myofiber type, mitochondrial biogenesis and oxidative capacity in the skeletal muscle, which collectively are linked with muscle insulin sensitization, are of great interest for informing therapeutic development in type II diabetes. ARNT may potentially have an important impact on myofiber type specification, mitochondrial biogenesis and glucose homeostasis via gene regulation. Therefore, this study was designed to investigate the role of ARNT in the skeletal muscle in the context of myofiber specification, mitochondrial biogenesis, oxidative capacity, obesity, and insulin resistance.

## Materials and Methods

### Mouse husbandry

Dr. Frank Gonzalez (NIH) generously provided ARNT floxed (ARNT^fl/fl^) mice. The targeting strategy for ARNT in these mice has been previously described [17], and the ARNT^fl/fl^ mice have been previously used to efficiently knockout ARNT in tissue-specific patterns in multiple studies [17]. We breed the ARNT^fl/fl^ mice with transgenic mice (Jackson labs, # 006149, B6.Cg-Tg (ACTA-cre)79Jme/) specifically over-expressing CRE recombinase enzyme in the skeletal muscle under the human skeletal actin (HSA) promoter. Briefly, after initial rounds of breeding using heterozygous mice, HSA-CRE::ARNT^fl/fl^ mice were cross bred with ARNT^fl/fl^ mice to obtained littermate ARNT^fl/fl^ (control) and HSA-CRE:: ARNT^fl/fl^ (MKO) mice. Both the HSA-CRE and ARNT^fl/fl^ mice were back-crossed for 2–3 generation to C57Bl/6J background before cross-breeding the two lines. The mice were bred and housed at the Brown Foundation Institute of Molecular Medicine’s vivarium. The room temperature was kept between 20–22°C under 12:12h light dark cycle with free access to water and food and were fed ad libitum on normal chow diet (NCD) (Pico Lab rodent diet 20; 13.2% Fat). For diet-induced obesity studies, mice were placed on a high fat diet (HFD) (Research Diet D12492, 60% Fat) starting at 7 weeks of age, for 14 weeks. All the experiments were performed in 3–7 months old mice. Within each experiment, all the groups were age and sex-matched. The exact age of the animals used in each experiment is stated in the figure legends. Cages were changed once a week. Animals were maintained and treated in accordance with the U.S. National Institute of Health Guide for Care and Use of Laboratory Animals, and the Animal Welfare Committee at The University of Texas McGovern Medical School at Houston approved all the procedures.

### Body mass and body composition

Body weight was measured weekly at the same time of the day. Body composition in terms of lean and fat mass was measured using quantitative nuclear magnetic resonance imaging (Echo-MRI 3-in-1 system; Echo Medical System).

### Tissue collection and preparation

Mice were euthanized under isoflurane anesthesia by cervical dislocation after 6 hr. of fasting and tissues were rapidly extracted. For RNA and protein, muscles were freeze-clamped in liquid nitrogen. For insulin signaling experiments, gastrocnemius muscles were stimulated in a KRHA buffer ± insulin (200 nM) *ex-vivo*, as previously described [18]. Because insulin-stimulated AKT-phosphorylation was measured in ex vivo experiments, we checked whether the quality of the gastrocnemius muscles across all groups is comparable by measuring ATP concentration in vehicle and insulin-stimulated muscles from control and MKO HFD-fed mice. ATP concentrations were measured using a luciferin/luciferase-dependent bioluminescence assay kit from Molecular Probes (Cat. # A22066), as per manufacturer’s instructions. Protein lystates from gastrocnemius muscles used for *ex vivo* measurement of insulin-stimulated AKT phosphorylation response test were subsequently used to measure ATP concentration. The assay was performed in 96 well plates and the luminescence was measured using Tecan 1000 plate reader in muscle lysates as well as ATP standards. The ATP concentrations in muscle lysates were determined from the ATP standard curve, normalized to protein concentrations of the muscle lysates, and presented as μmol ATP/g.

For immunofluorescence experiments, Tibialis Anterior (TA) muscles were mounted in OCT and frozen in melting isopentane cooled down by liquid nitrogen.

### Gene expression

Total RNA was prepared using the Purelink Kit (Ambion, Life technologies). Total RNA was further reverse-transcribed to cDNA with SuperScript III Reverse Transcriptase (Invitrogen) and analyzed by quantitative real-time PCR using ABI-7900 cycler (Applied Biosystems). The genes were normalized to *Ppia* for the tissues or *Hprt* for the myotubes. List of primers used and sequences is provided (S2 Table).

### Protein analysis by western blotting

Tissues were homogenized in Pierce IP Lysis buffer (Thermo Scientific) using a Polytron instrument at 25,000 rpm. Further the lysates were pre-cleared at 16,000 x g for 20 min at 4°C, and the supernatants were store at -80°C. The protein content was measured using the Pierce BCA protein assay kit (Thermo Scientific). Protein samples (30–40 μg) were run on an 8 or 10% or 4–20% (VWR) poly-acrylamide gel, transferred onto nitrocellulose membranes and incubated with the primary antibodies [anti-pAKT ser-473 (# 4058), anti-panAKT (# 4691), ARNT (# 5537) (Cell Signaling Technology), OXPHOS cocktail (# ab110413 (Abcam), GLUT4 (# bs1658R (Bioss)].

### Histological analysis

The H&E staining was performed on serial transverse cryosections (10 μm thickness) obtained from the mid-section of the TA muscles. After fixation with a 4% PFA/PBS solution, slides were incubated in hematoxylin QS (Vector Laboratories) for 2 minutes followed by 5 min washing under running tap water. Following 1 min incubation with a 90% ethanol solution, the slides were incubated for 2 minutes in the Eosin solution (VWR). Prior to mounting with Permount (Fisher scientific) slides were dehydrated by successively incubation in 70%, 90%, 100% EtOH and 100% Xylen solution.

### Immunohistology

Serial transverse cryosections (10 μm thickness) were obtained from the mid-section of the TA muscles. Frozen muscle sections were processed for immunofluorescence staining of capillaries [using CD31/PECAM-1 antibody # RM5200 (Invitrogen)] and of myosin heavy chains (MyHC) type IIA, IIX and IIB [using, respectively, # SC-71, # BF-F3, and # 6H1 (Developmental Studies Hybridoma Bank)], as we previously described [19,20]. All primary antibodies were visualized using suitable Alexa Fluor® or Cy5 secondary antibodies from Molecular Probes. Negative control staining by omitting either the primary or the secondary antibody was included in all sets of experiments. In addition, we used microsphere (Invitrogen) angiography for detecting intact muscle vascular supply, as we previously described [15].

### Succinate Dehydrogenase (SDH) and Nicotinamide Adenine Dinucleotide-Tetrazolium Reductase (NADH-TR) Staining

SDH and NADH-TR staining was performed on cryosections (10 μm thickness) of TA muscles, as previously described [21,22].

### Insulin and glucose tolerance test

Tests were performed as previously described [23]. Insulin (Sigma-Aldrich) was injected at the dose of 0.4 U/kg for the Insulin tolerance test (ITT). For the glucose tolerance test (GTT), we injected 2 g/kg or 1 g/kg of glucose, respectively, for NCD and HFD-fed mice. Area under the curve (AUC) or area above the curve (AAC) was calculated to account for differences in baseline fasting blood glucose concentrations, as previously described [24]. Note that the NCD and HFD studies were performed as independent experiments, such that the mice in NCD cohort were at a different age than the HFD cohort. Therefore, the effect of diet is not being examined by comparing ITT and GTT data from the NCD and HFD cohorts on the same graph.

### Blood Parameters

After 6 hr. of fasting, blood was collected in EDTA-coated tubes and then centrifuged at 2000 x g for 10 min at 4°C. The plasma glucose and insulin levels were measured using a colorimetric assay (Cayman Biochemicals) and an ELISA kit (Alpco), respectively. The Quicki index was calculated using the glucose and insulin values, as we previously described [25].

### Primary culture

Primary muscle cells were isolated from the ARNT^fl/fl^ mice, as previously described [26]. Briefly, the hind limb muscles were digested in a Collagenase D/Dispase/CaCl2 cocktail solution (0.345 U/ml, 2.4 mg/ml and 2.5 mM, respectively). Subsequently, the myoblasts were isolated using the pre-plating technique. For the experiments, the primary cells were seeded on matrigel-coated plates. At 80% confluence the medium was changed from growth (F10 medium supplemented with 20% FBS and 2.5ng/ml bFGF) to the differentiation medium (DMEM 10% Horse serum), and the cells were infected with control (Ad-control) or CRE (Ad-CRE) adenoviruses (Vector Development Lab, Baylor College of Medicine) at the MOI of 100 for 48 hr. After 5 days of differentiation the cells were treated with DMOG (1 mM) or DMSO (vehicle) for 16 hr., and subsequently harvested for QPCR analysis.

### Statistical analysis

Statistical analysis was performed using GraphPad Prism 5.0 for Windows (GraphPad Software Inc.). Normal distribution of the data was tested with Kolmogorov-Smirnov test. Unpaired Student’s t-tests with or without Welch correction were performed to determine differences between two groups. One-way ANOVA with Tukey’s multiple comparison test was used for comparing more than two groups. Two-way ANOVA with Bonferroni’s repeated measure test was used for comparing groups when two or more variables were present. All values in figures are presented as mean ± SEM. Statistical significance was set at p<0.05.

## Results

### ARNT is preferentially expressed in the oxidative slow-twitch skeletal muscle

**Fig 1:**
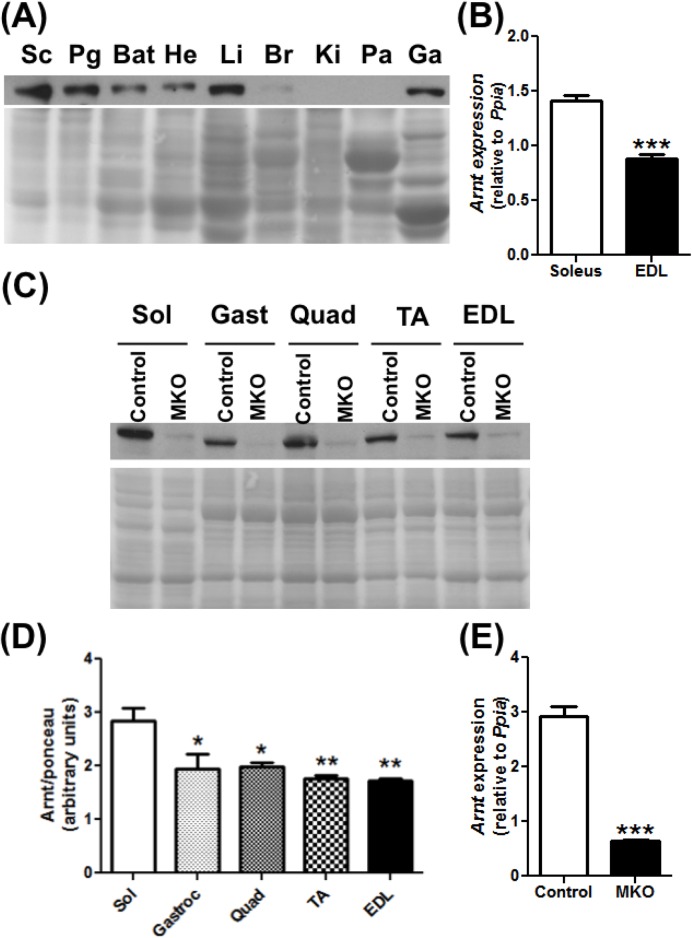
ARNT expression in different tissues. **(A)** ARNT protein expression in different organs [sub-cutaneous adipose tissue (Sc), perigonadic adipose tissue (Pg), brown adipose tissue (Bat), heart (He), liver (Li), brain (Br), kidney (Ki), pancreas (Pa), gastrocnemius muscle (Ga)] of 4 months old mice (N = 1). **(B)** Arnt gene expression in the soleus and the extensor digitorum longus (EDL) of 3 month old mice (N = 4–5). **(C-D)** ARNT expression in control and MKO muscle groups ranging from the most oxidative (soleus) to most glycolytic (EDL) (N = 3). (C) Representative images. (D) Densitometry for protein expression. (E) Arnt gene expression in EDL control and MKO muscles of 4 months old mice (N = 4–5). (*p<0.05,**p<0.01,***p<0.001, Unpaired Student’s t-test or One-way ANOVA with Tukey’s multiple comparison post-hoc test.)

We first measured the expression of ARNT protein in skeletal muscles relative to various other organs. ARNT is highly expressed in adipose tissues, heart, liver, and also skeletal muscle (Fig 1A). We further measured whether there is any difference in ARNT expression between the pre-dominantly oxidative slow-twitch muscles and the pre-dominantly glycolytic fast-twitch muscles, keeping in mind that oxidative slow-twitch muscles are metabolically efficient and relatively more insulin sensitive [27,28]. Expression of both ARNT gene (Fig 1B) and protein (Fig 1C and 1D) was relatively higher in pre-dominantly oxidative muscle (e.g. soleus) compared to the glycolytic muscles (e.g. gastrocnemius, quadriceps, TA, EDL). Nevertheless, ARNT is robustly expressed in both types of muscle beds.

### Characterization of the MKO mice

To specifically study the effect of ARNT in the skeletal muscle, we generated mutant (MKO) mice in which ARNT is selectively deleted in the skeletal muscles. In these mice, *cre-*driven recombination in the skeletal muscle results in a 79% decrease in *Arnt* gene expression in the EDL (Fig 1E) and a 74% decrease in the soleus (S1A Fig). The ARNT protein expression is severely blunted in the skeletal muscles of the MKO mice, as exemplified in different hindlimb muscles (Fig 1C). The loss of muscle ARNT expression is not compensated by an increase in the expression of *Arnt2* or *Arnt3*, which are the other two isoforms of ARNT (data not shown). As expected from the use of muscle-specific CRE, ARNT expression was not deleted in the other organs of the MKO mice (S1B Fig). In additional in vitro experiments performed in myocytes isolated from the skeletal muscles of ARNT^fl/fl^ mice, we demonstrate that deletion of ARNT ablates DMOG (hypoxia mimetic/HIF stabilizer drug)-mediated induction of HIF targets genes (*Slc2a1*, *Vegfa*, *Ldha*) (S2 Fig). This confirms that the deletion of ARNT leads to suppression of its transcriptional activity associated with the HIF pathway. We do not understand why the baseline expression of ARNT is decreased by DMOG, but perhaps it’s a compensatory response to HIF1 activation by DMOG.

**Fig 2:**
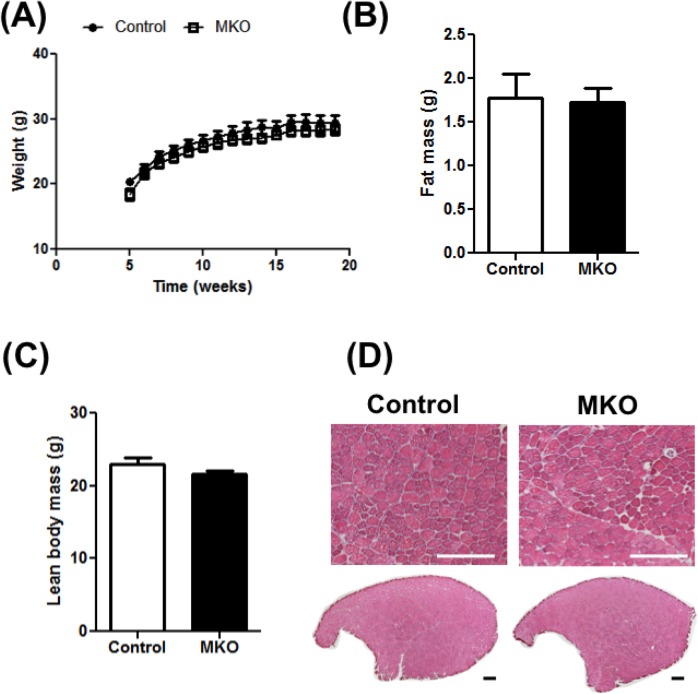
Characterization of the MKO mice. Following parameters were measured in wild type control and MKO mice. **(A)** Weight gain measured from 5 to 20 weeks of age (N = 10–12). **(B-C)** Body composition measured as fat mass (B) and lean mass (C) (N = 4). (**D**) H&E staining showing histomorphology of the tibialis anterior (TA) muscle cross-sections (N = 4). (Scale bar = 500 μm.) (***p<0.001, Unpaired Student’s t-test.)

We first characterized the weekly changes in body weight of the MKO mice from 5 weeks to 20 weeks of age. The initial weight as well as age-dependent weight gain in MKO mice is comparable to the control littermate mice (Fig 2A). We also measured the total body fat and the lean body mass, using Echo-MRI, in 4 months old animals and did not find any difference between the control and the MKO mice (Fig 2B and 2C). In confirmation of the Eco-MRI data, the individual weights of different muscles, as well as the perigonadic and the brown adipose tissue were comparable between the control and MKO mice (S1 Table). Furthermore, in cross-sections of TA muscles stained with H&E, we did not find any difference in myofiber size and number between control and MKO mice (Fig 2D).

### Muscle-specific ARNT deletion does not affect oxidative and vascular phenotype in the skeletal muscle

**Fig 3:**
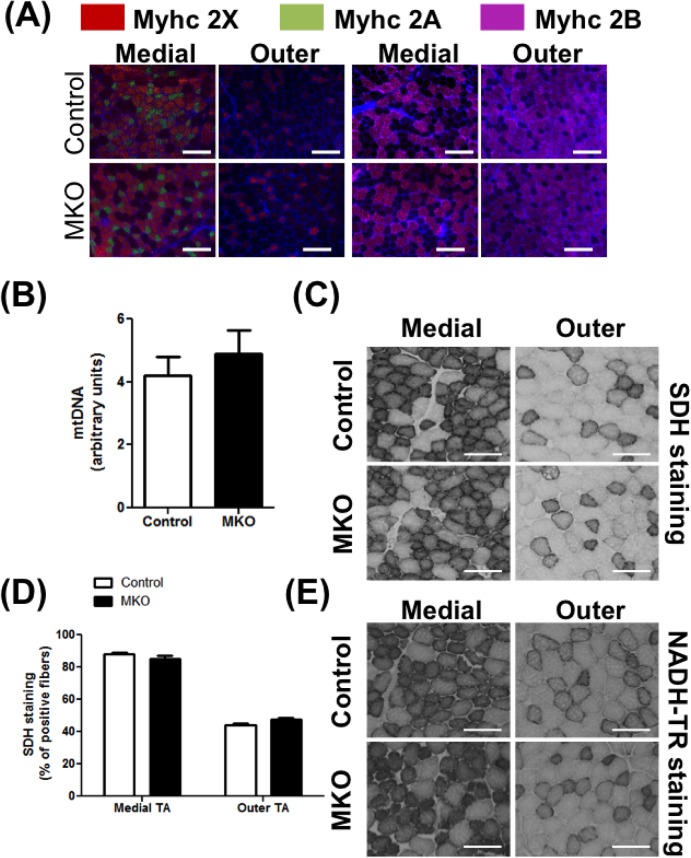
Fiber type and oxidative capacity in NCD-fed mice. Following parameters were measured in **4 months old** control and MKO mice on NCD. **(A)** Representative images of medial (left) and outer (right) TA cross-sections stained for MyHC IIA (green), IIX (red), and IIB (purple) (N = 4–6). **(B)** Mitochondrial DNA content in EDL (N = 4–6). **(C-D)** Succinate dehydrogenase (SDH) staining activity in cross-sections of TA. **(C)** Representative cross-sectional images of the outer and medial TA (N = 4–6). **(D)** Percentage of positive fibers quantified in the outer and medial TA cross-sections for the SDH staining (N = 4–6). **(E)** Representative cross-sectional images of the outer and medial TA with NADH-TR staining (N = 4–6). (Scale bar = 200 μm.) The differences between groups were not statistically significant (Unpaired Student’s t-test).

The relatively high expression of ARNT gene and protein in predominantly oxidative slow-twitch vs. the glycolytic fast-twitch muscle lead us to measure the effect of muscle ARNT deletion on myofiber specification. We measured the expression of *MyHC 7*, *2*, *1* and *4* genes (respectively encoding MHC I, IIA, IIX and IIB proteins representing the namesake myofiber types) in the quadriceps of the control and MKO mice. No differences were observed in the expression of the myofiber biomarker genes between the two groups (S3A Fig). We also measured myofiber type composition by immunofluorescence, but no fiber type switch was observed in the TA of the MKO mice compared to their control littermates (Fig 3A). We then assessed the mitochondria content in the EDL using the mitochondrial DNA as read out, and also did not detect any change between control and MKO mice (Fig 3B). In support, we did not find any difference in the expression of protein markers of mitochondrial respiratory complex (S4A). The maximal mitochondrial activity measured by the SDH (complex II) staining of the muscle cryosections between the two genotypes (Fig 3C and quantified in Fig 3D), as well as by NADH-TR (complex I) staining (Fig 3E) was comparable between the control and the MKO mice. We also measured the expression of key nuclear receptors (*Esrra*, *Esrrg*, *Ppara*, *Ppard*, *Ppargc1a*, *Ppargc1b*) that regulate mitochondrial biogenesis and lipid oxidation, and were previously described to be ARNT targets in non-muscular tissues [5,12,29]. The expression of these genes was unaffected by ARNT deletion in the skeletal muscle (S5A Fig). Likewise, the expression of majority of genes directly encoding features of oxidative myofibers were unaffected by ARNT deletion (*Acadm*, *Cd36*, *Cpt1b*, *Ckmt2*, *Mb*, *Pdhk4*, *Tnni1*) (S5B Fig).

As another marker of the oxidative muscles, we also measured the total capillary density as well as the functional vascular supply in the aforementioned muscles. Typically, oxidative muscles are highly vascularized. We did not find any change in CD31-positive capillary staining (S6A Fig) or in the density of microsphere-perfused blood vessels (S6B Fig) in the TA muscles of MKO compared to the control mice. In agreement with these results, the expression of HIF-targeted angiogenic factors such as *Vegfa121*, *Vegfa165* and *Vegfa189* did not change in the TA muscles of MKO compared to the control mice (S6C Fig).

**Fig 4:**
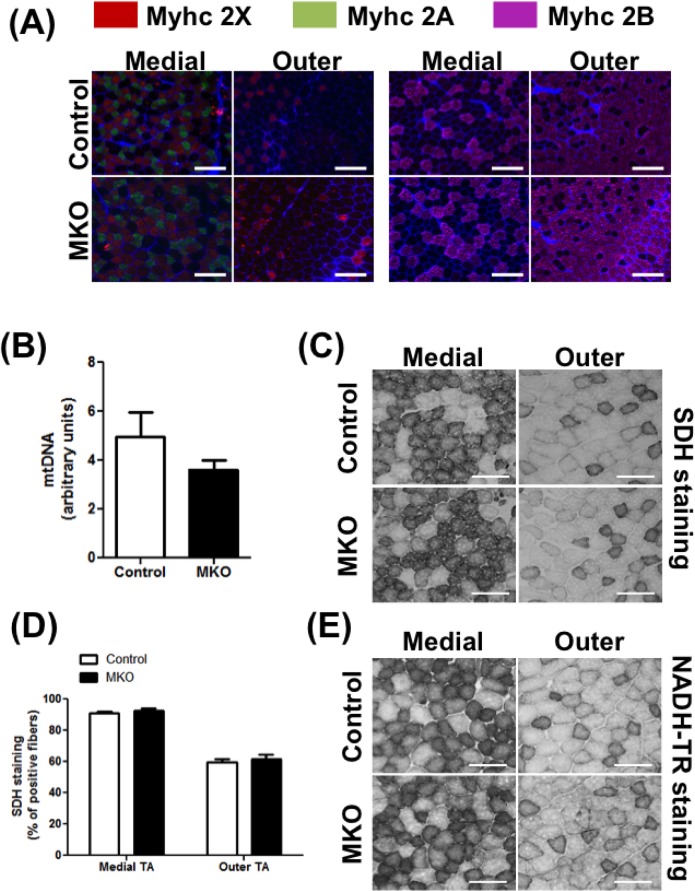
Fiber type and oxidative capacity in HFD-fed mice. Following parameters were measured in 5 months old control and MKO mice on HFD. **(A)** Representative images of medial (left) and outer (right) TA cross-sections stained for MyHC IIA (green), IIX (red), and IIB (purple) (N = 6–7). **(B)** Mitochondrial DNA content measured in EDL (N = 6–7). **(C-D)** SDH staining activity in TA. **(C)** Representative images of the outer and medial TA muscle (N = 6–7). **(D)** Percentage of positive myofibers measured in the outer and medial TA for the SDH staining (N = 6–7). **(E)** Representative images of the NADH-TR staining activity in the outer and medial TA muscle (N = 6–7). (Scale bar = 200 μm.) The differences between groups were not statistically significant (Unpaired Student’s t-test).

It is well established that high fat diet (HFD) fed mice adapt by increasing muscle oxidative capacity [30] in an attempt to combat obesity and insulin resistance. ARNT could be responsible for oxidative myofiber remodeling under obesity-inducing dietary condition, which is what we tested. Both the control and the MKO mice were placed on 60% Kcal high fat diet for 14 weeks, and subsequently following measurement were made. Firstly, we compared the muscle oxidative capacity in the HFD-fed mice and normal chow diet (NCD)-fed mice, as measured by mitochondrial DNA content, SDH staining and NADH-TR staining. We did not observed an increase of the mitochondrial DNA expression (NCD = 4.19±0.6 and HFD = 4.95±1.01, p = NS) in skeletal muscles of HFD-fed vs. the NCD-fed mice. However, the SDH and NADH-TR activities were higher in the skeletal muscle of HFD-fed mice compared to the NCD-fed mice (S7A–S7D Fig). Despite the oxidative remodeling observed in the muscle after HFD, deletion of ARNT expression in the skeletal muscle did not affect mitochondrial DNA content (Fig 4B), mitochondrial complex density (S4B Fig) and SDH (Fig 4C and 4D) or NADH-TR (Fig 4E) activity, in HFD-fed MKO mice compared to the littermate control mice. As in the NCD-fed mice the expression of the *MyHC* genes (S3B Fig) and the fiber type (Fig 4A) distribution was not altered in the HFD-fed MKO mice compared to the HFD-fed littermate controls. Of note, some of the known HIF target genes were induced by HFD feeding (Hk1 and Glut1) in the muscle, but the induction was not affected by ARNT deletion (data not shown).

Altogether these results show that deletion of ARNT in the skeletal muscle does not affect myofiber type composition, mitochondrial content or oxidative capacity in the skeletal muscle either under NCD or HFD-feeding conditions.

### Muscle-specific ARNT deletion does not affect HFD-induced weight gain and insulin resistance

**Fig 5:**
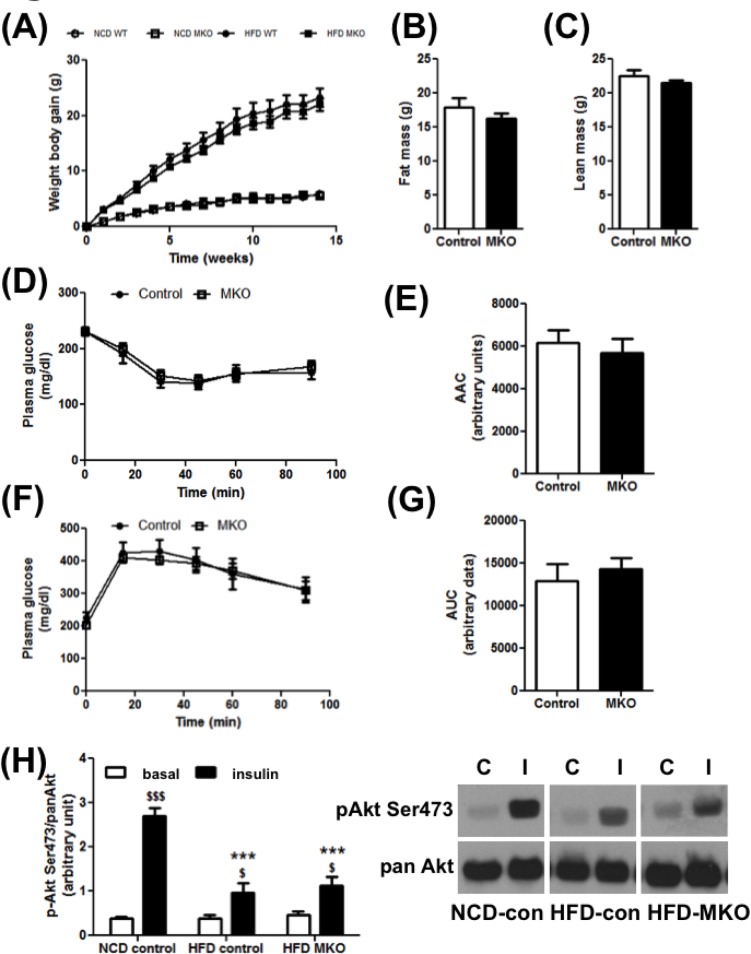
Insulin and Glucose Tolerance. Following parameters were measured in control and MKO mice on HFD. (A) Weight gain (N = 6–12). (B) Fat mass in 4 month old mice (N = 6–12). (C) Lean mass in 4 month old mice (N = 6–12). (D) Insulin tolerance test (ITT) in 4 months old mice (N = 6–11). (E) Area Above the Curve (AAC) for ITT. (F) Glucose tolerance test (GTT) in 4 months old mice (N = 6–11). (G) Area Under the Curve (AUC) for GTT. (H) Ex vivo p-AKT ser473/panAKT stimulation by insulin measured in the gastrocnemius muscles of the 5 months old mice (N = 3–5). $ indicates the treatment effect; * indicate the genotype effect. $ p<0.05; $ $ $/*** p<0.001 (Unpaired Student’s t-test or Two-way ANOVA with a Bonferroni’s repeated measure test).

Twelve weeks of HFD feeding lead to a significantly greater increased in weight gain compared to NCD feeding in mice. However, we did not detect any significant difference in weight gain between HFD-fed MKO and littermate control mice (Fig 5A). The respective weights in control and MKO mice at the end of 12 weeks of HFD were 45.3±1.8 g and 43.13±1.3 g. The lean and fat mass measured by Eco-MRI was not altered in the MKO compared to the littermate control mice (Fig 5B and 5C). HFD resulted in an increase in plasma glucose (S8A Fig) and insulin (S8B Fig) levels compared to the NCD. Calculation of QUICKI index, which is a measure of insulin sensitivity indicated 12 weeks of HFD resulted in insulin resistance (S8C Fig). We next measured the insulin sensitivity of the animals after 10 weeks of HFD. We did not observe any difference in plasma glucose levels during the insulin tolerance test (ITT) (Fig 5D and 5E) or glucose tolerance test (GTT) (Fig 5F and 5G) between the two genotypes. Note that the insulin sensitivity and glucose tolerance in MKO mice were also similar to the control littermate mice on NCD (S9A and S9B Fig).

To assess muscle insulin signaling we performed an *ex vivo* insulin stimulation assay on the isolated gastrocnemius muscles from different mice and measured AKT phosphorylation on the activating serine 473 residue. While insulin stimulated AKT phosphorylation in *ex vivo*-treated gastrocnemius muscle from NCD-fed mice, this effect was impaired in HFD-fed mice. Furthermore in the HFD-fed group, there was no difference in the level of AKT phosphorylation at baseline or under insulin-stimulated conditions between the control and the MKO groups, after normalization by the total protein (Fig 5H, *Left panel*: Densitometry ratio; *Right panel*: Representative blots). To ensure that the quality of isolated muscles used in insulin-stimulated AKT phosphorylation is comparable between groups, we measured ATP concentrations in muscle lysates from different groups. The ATP levels in all the muscle groups were found to be similar [HFD-control vehicle-treated: 0.0059±0.002 μmoles ATP/g, HFD-control insulin-treated: 0.0053±0.0013 μmoles ATP/g, HFD-MKO vehicle-treated: 0.00453±0.00032 μmoles ATP/g, HFD-MKO Insulin-treated: 0.0065±0.0016 μmoles ATP/g, p = NS, One-way ANOVA with Tukey’s multiple comparison post-hoc test]. The gene expression of glucose transporters GLUT1 (*Slc2a1*) and GLUT4 (*Slc2a4*), which were previously identified as targets of ARNT in the adipocytes [11] are not affected by the deletion of ARNT in the skeletal muscle, either in NCD (S10A Fig) or HFD-fed (S10B Fig) mice. We did not observe any change in the protein expression of the GLUT4 transporter either in NCD or HFD groups (S10C and D). Therefore, unlike ARNT in other metabolically relevant organs, skeletal muscle ARNT expression does not influence the progression of obesity and insulin resistance.

## Discussions

Here we show that ARNT is expressed in the skeletal muscle, and its expression is relatively higher in oxidative slow-twitch muscles compared to the glycolytic fast-twitch muscles. Despite its robust expression in the skeletal muscle, deletion of ARNT selectively in the muscle does not affect myofiber type composition, mitochondrial biogenesis and oxidative capacity in the muscle. Unlike its role in the other tissues, deletion of muscle ARNT does not affect weight gain or pathogenesis of insulin resistance and glucose intolerance in murine model of diet-induced obesity.

Despite the strong rationale presented in the *Introduction*, deletion of ARNT in the skeletal muscle did not have any effect on the proportions of different myofibers and related factors such as the level of mitochondrial biogenesis and oxidative capacity. The lack of effect of ARNT knockout is especially surprising in the context of the role of HIF1A and HIF2A in the skeletal muscle, both of which require obligatory heterodimerization with ARNT to be functional [1]. In most other organ systems cellular functions of ARNT vs. HIF1A or HIF2A are generally overlapping [11,17,29]. This could suggest that in muscle HIF1A and HIF2A may function by heterodimerizing with other transcriptional factors in absence of ARNT. As much as two other isoforms of ARNT, namely ARNT 2 and 3 (also known as Bmal1) have been discovered, whether they can serve as HIF heterodimer partners remains unknown. We did not find any compensatory increase in ARNT 2 or 3 expression in the muscles lacking ARNT. Further, in primary myocytes isolated from ARNT^fl/fl^ mice, we found that ARNT is indispensible for induction of HIF target genes by a HIF stabilizer/hypoxia mimetic drug DMOG, supporting the likelihood that the lack of effect of ARNT on fiber type is unrelated to substitution by a surrogate protein or transcriptional factor.

High fat feeding, leading to obesity and insulin resistance brings about a compensatory remodeling of the skeletal muscle towards an oxidative phenotype to cope with excessive energy and lipid accumulation, under which several muscle signaling pathways become relevant [30–32]. Indeed, we showed that HFD feeding leads to a compensatory increase in oxidative capacity in the skeletal muscle. However, even under HFD feeding conditions, lack of muscle ARNT expression does not impact the aforementioned adaptation or as such change myofiber type composition, oxidative capacity, mitochondrial content, weight gain and insulin tolerance. The effect of HFD feeding on muscle capillarity in mice is unclear, as in one study HFD caused muscle capillary regression in mice [33]; whereas, in another it increased angiogenesis and capillarity, without affecting HIF1 expression [34]. Nevertheless, ARNT deletion did not affect capillary density at least in the skeletal muscles of NCD-fed mice, which was somewhat surprising given the central role for HIF1 in regulating angiogenesis. Furthermore, ARNT did not seem to affect the expression of any genes relevant to aforementioned muscle remodeling processes, including regulators of oxidative metabolism (e.g. *Essra*, *Essrg*, *Ppargc1a*, *Ppargc1b*, *Ppara*, *Ppard*), MyHC isoforms, other metabolic markers of oxidative muscles (*Acadm*, *Cd36*, *Cpt1b*, *Ckmt2*, *Mb*, *Pdhk4*, *Tnni1*), and angiogenic factors (*Vegfa121*, *Vegfa165* and *Vegfa189*). This is in sharp contrast to the transcriptional effects of ARNT in other tissues through which it regulates energy homeostasis. In liver, ARNT seems to maintain energy and glucose homeostasis via the suppression of gluconeogenesis and lipid metabolism genes primarily through regulation of master-regulators such as FXR and SREBP-1c [12]. In adipose tissue, ARNT positively controls genes linked with angiogenesis (*Vegf*), and thus its deletion prevents adipose expansion and glucose intolerance [11]. However, ARNT deletion decreases the glucose uptake capacity of adipocyte by down-regulating glucose transporters 1 and 4. In pancreatic beta cells, ARNT encodes the expression of genes linked with glucose metabolism and insulin signaling, which ultimately affects insulin secretion by these cells [6].

What might be the role of ARNT in the skeletal muscle? Skeletal muscle remodeling occurs under a variety of conditions including exercise, inactivity, ischemic muscle angiopathy, aging/sarcopenia and muscle regeneration. HIF1A and HIF2A signaling is important in many of these conditions. For example, HIF1A has been described to be essential in skeletal muscle adaptation to endurance type exercise [35,36]. In gain-of-function studies, both HIF1A and HIF2A activation in the skeletal muscle can induce angiogenesis and revascularization in murine models of hind limb ischemia [37–42]. Recent studies have also implicated both HIF1A and ARNT in satellite cell activation and regeneration in muscle injury [43]. Therefore, ARNT could still potentially be involved in muscle remodeling in any of these conditions. Moreover, ARNT interacting partners may not be limited to HIF family members, and therefore, ARNT could influence gene clusters unrelated to metabolism and fiber type in the skeletal muscle. Identification of these gene-sets in future will require global gene expression analysis and/or ChIP-sequencing analysis comparing control and ARNT null skeletal muscles. Further, identification of transcriptional protein complexes containing ARNT in the skeletal muscle using mass-spectrometry analysis will provide additional insights into the ARNT transcriptional role in the muscle. Finally, muscle-specific gain-of-function mouse models may reveal muscle ARNT function that may be masked by gene redundancy.

In conclusion we show that muscle-specific ARNT deletion does not affect myofiber distribution, mitochondrial content and oxidative capacity despite being highly expressed in oxidative slow-twitch muscles. Furthermore, ARNT deletion also does not affect skeletal muscle mass, weight body gain, or insulin/glucose homeostasis in animals on either normal chow or high fat diet. Overall, muscle ARNT expression is dispensable for metabolic regulation and energy homeostasis.

## Supporting Information

S1 Fig Arnt expression. (A) *Arnt* gene expression in the soleus of 4 months old control and MKO mice (N = 4–5). (B) ARNT protein expression in the perigonadic adipose tissue (PG), heart and liver of 3 months old WT and MKO mice (N = 2). (***p<0.001, Unpaired Student’s t test.)
(TIFF)

S2 Fig HIF1 target gene expression in myoblast. Gene expression measured in primary wild type myoblasts treated with Ad-control or Ad-Cre, and with DMOG (N = 3) (1uM) or DMSO as control. (A) *Arnt* expression. (B) *Slc2a1* expression. (C) *Vegfa* expression. (D) *Ldha* expression. * Indicates a treatment effect and $ indicates gene knockout effect. $ $ p<0.01; $ $ $/*** p<0.001 (Two-way ANOVA with a Bonferroni’s repeated measure test).
(TIFF)

S3 Fig MyHC gene expression. **(A-B)** *MyHC* gene expression in the skeletal muscles of NCD (A) and HFD-fed (B) 5 months old control and MKO mice (N = 5–6). (p = NS, Unpaired Student’s t-test.)
(TIFF)

S4 Fig Mitochondrial complex protein expression. **(A-B)** Expression of mitochondrial complex proteins in the skeletal muscles of NCD (A) or HFD-fed (B) 5 months old mice. Representative images from N = 4–6 per group.
(TIFF)

S5 Fig Nuclear receptor and transcriptional factor expression in muscle. **(A-B)** Gene expression of nuclear receptors and transcription factors (A) and their known target genes (B) in the skeletal muscle of 5 months old NCD-fed mice (N = 4–6). (p = NS, Unpaired Student’s t-test.)
(TIFF)

S6 Fig Microvascular staining in muscle. (A) Immunostaining for the endothelial marker CD31/PECAM-1 in the TA muscle cross-sections of 7 months old control and MKO mice (N = 4). (B) Representative cryo-section images of TA muscles from 7 months old control and MKO mice, perfused with fluorescent microspheres (N = 4). (C) Gene expression of *Vegfa* isoforms (N = 8–7). (Scale bar = 200 μm). (p = NS, Unpaired Student’s t-test.)
(TIFF)

S7 Fig SDH and NADH-TR staining. **(A-B)** SDH activity staining in TA muscle cross-sections from 4–5 months old NCD and HFD-fed mice (N = 5–6). **(A)** Representative images of the outer and medial TA muscle. **(B)** Percentage of SDH positive myofibers. **(C-D)** NADH-TR activity staining in TA muscles of 4–5 months old NCD and HFD-fed mice (N = 6). **(C)** Representative images of the outer and medial TA muscle. **(D)** Percentage of NADH-TR positive myofibers. (Scale bar = 200 μm). (**p<0.01, ***p<0.001, Unpaired Student’s t test.)
(TIFF)

S8 Fig Fasting plasma glucose and insulin. **(A-B)** Fasting plasma glucose (A) and insulin (B) levels in 6 hr. fasted 5 months old control and MKO NCD or HFD-fed mice (N = 5–6). (C) Quicki index in 5 months old control and MKO NCD or HFD-fed mice (N = 5–6). *Indicates a diet effect. **p<0.01 *** p<0.001 (Two-way ANOVA with a Bonferroni’s repeated measure test).
(TIFF)

S9 Fig GTT and ITT in NCD-fed mice. **(A-B)** Insulin tolerance test **(A)** and Glucose tolerance test **(B)** in 6 hr. fasted 3 months old control and MKO NCD-fed mice (N = 6–7). (p = NS, Two-way ANOVA with a Bonferroni’s repeated measure test.)
(TIFF)

S10 Fig Glucose transporter expression in muscle. The following parameters are measured in 6 hr. fasted 5 months old control and MKO NCD or HFD-fed mice. **(A)** Muscle gene expression of glucose transporters (*Slc2a1*, *Slc2a4*) in NCD-fed mice (N = 4–6). **(B)** Muscle gene expression of glucose transporters in HFD-fed mice (N = 5–6). **(C)** Muscle GLUT4 protein expression in NCD-fed mice (N = 4–6). **(D)** Muscle GLUT4 protein expression in HFD-fed mice (N = 5). (p = NS, Unpaired Student’s t-test.)
(TIFF)

S1 Table Tissue weights in 4 month-old male control and MKO mice. (PDF)

S2 Table QPCR primer sequences. (PDF)
