# Supplementary figures and images for: Muscle Arnt/Hif1β Is Dispensable in Myofiber Type Determination, Vascularization and Insulin Sensitivity
Source: PLoS One. 2016 Dec 22;11(12):e0168457. doi: 10.1371/journal.pone.0168457 (PMC5178999; doi:10.1371/journal.pone.0168457)

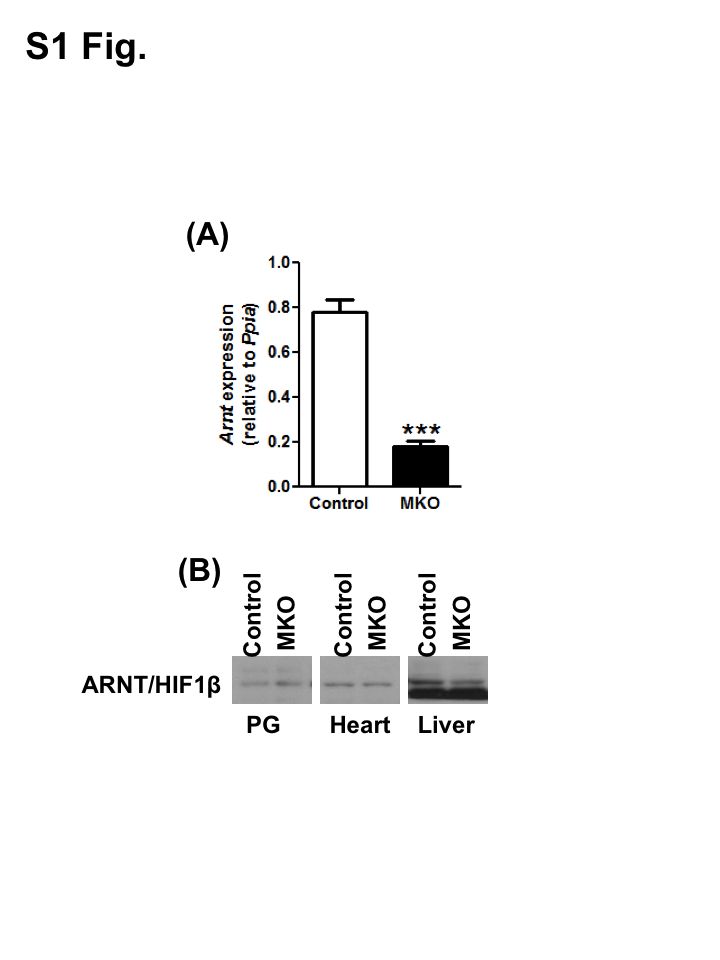

Supplement: S1 Fig — (A) Arnt gene expression in the soleus of 4 months old control and MKO mice (N = 4–5). (B) ARNT protein expression in the perigonadic adipose tissue (PG), heart and liver of 3 months old WT and MKO mice (N = 2). (***p<0.001, Unpaired Student’s t test.) (TIFF) [file pone.0168457.s001.tiff]

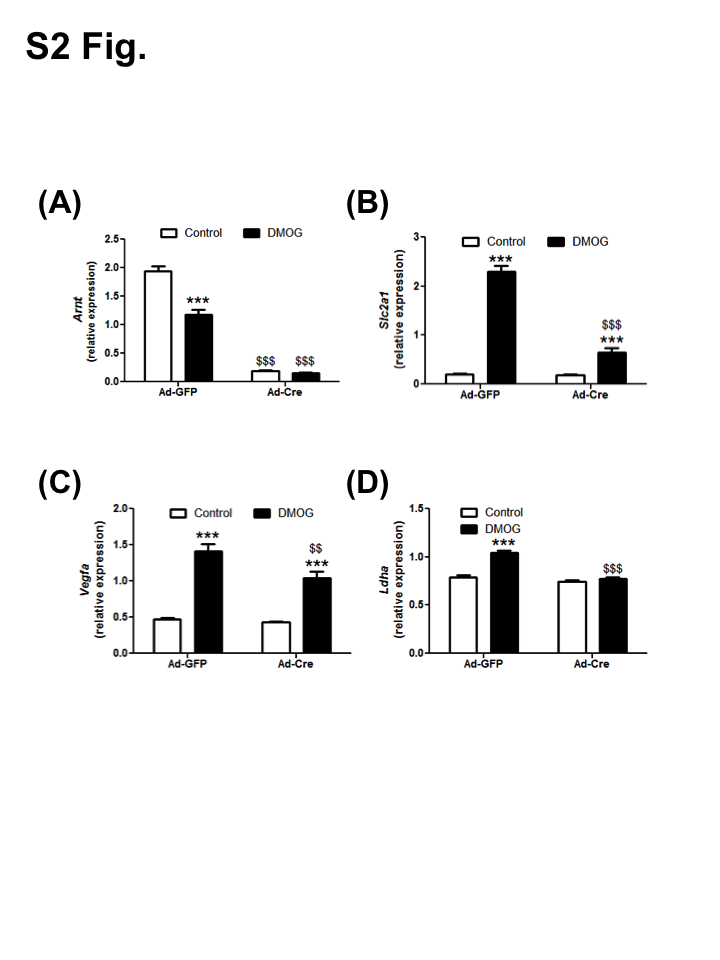

Supplement: S2 Fig — Gene expression measured in primary wild type myoblasts treated with Ad-control or Ad-Cre, and with DMOG (N = 3) (1uM) or DMSO as control. (A) Arnt expression. (B) Slc2a1 expression. (C) Vegfa expression. (D) Ldha expression. * Indicates a treatment effect and $ indicates gene knockout effect. $ $ p<0.01; $ $ $/*** p<0.001 (Two-way ANOVA with a Bonferroni’s repeated measure test). (TIFF) [file pone.0168457.s002.tiff]

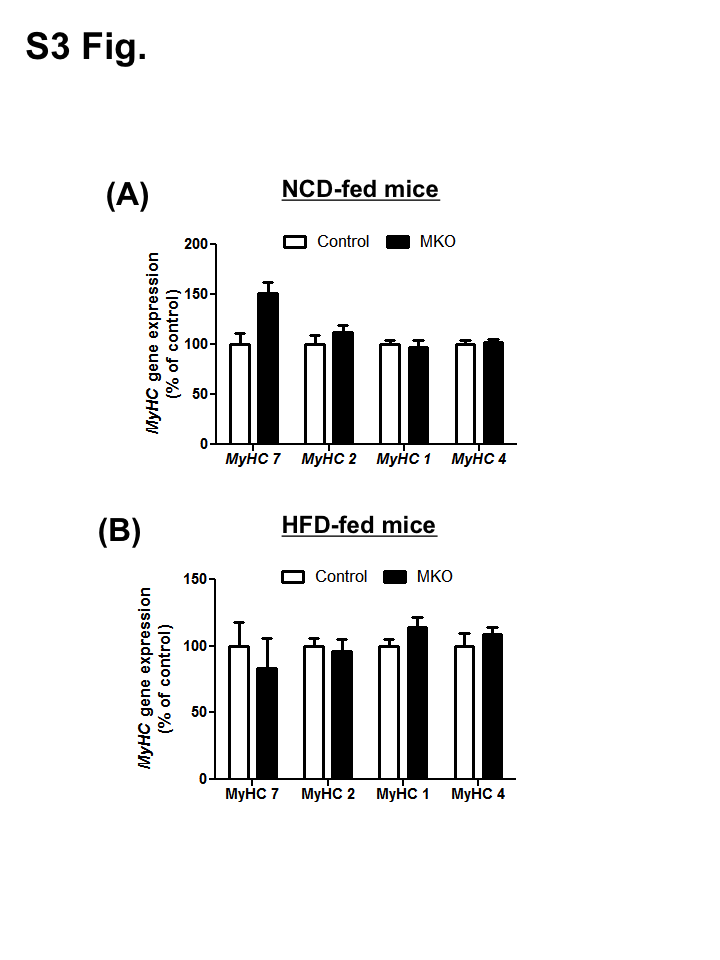

Supplement: S3 Fig — (A-B) MyHC gene expression in the skeletal muscles of NCD (A) and HFD-fed (B) 5 months old control and MKO mice (N = 5–6). (p = NS, Unpaired Student’s t-test.) (TIFF) [file pone.0168457.s003.tiff]

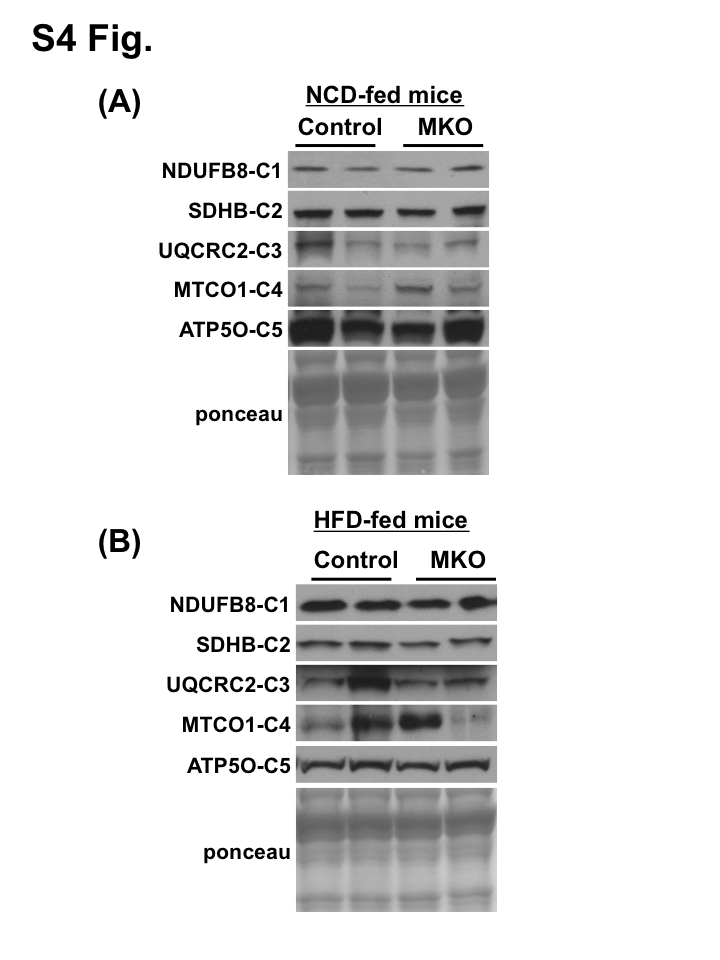

Supplement: S4 Fig — (A-B) Expression of mitochondrial complex proteins in the skeletal muscles of NCD (A) or HFD-fed (B) 5 months old mice. Representative images from N = 4–6 per group. (TIFF) [file pone.0168457.s004.tiff]

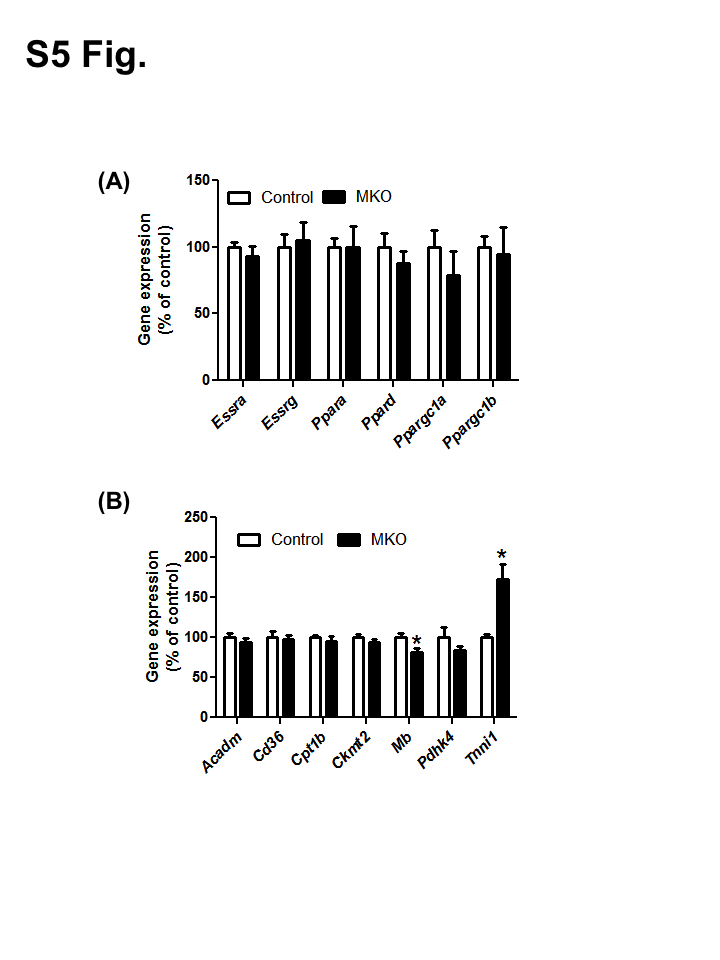

Supplement: S5 Fig — (A-B) Gene expression of nuclear receptors and transcription factors (A) and their known target genes (B) in the skeletal muscle of 5 months old NCD-fed mice (N = 4–6). (p = NS, Unpaired Student’s t-test.) (TIFF) [file pone.0168457.s005.tiff]

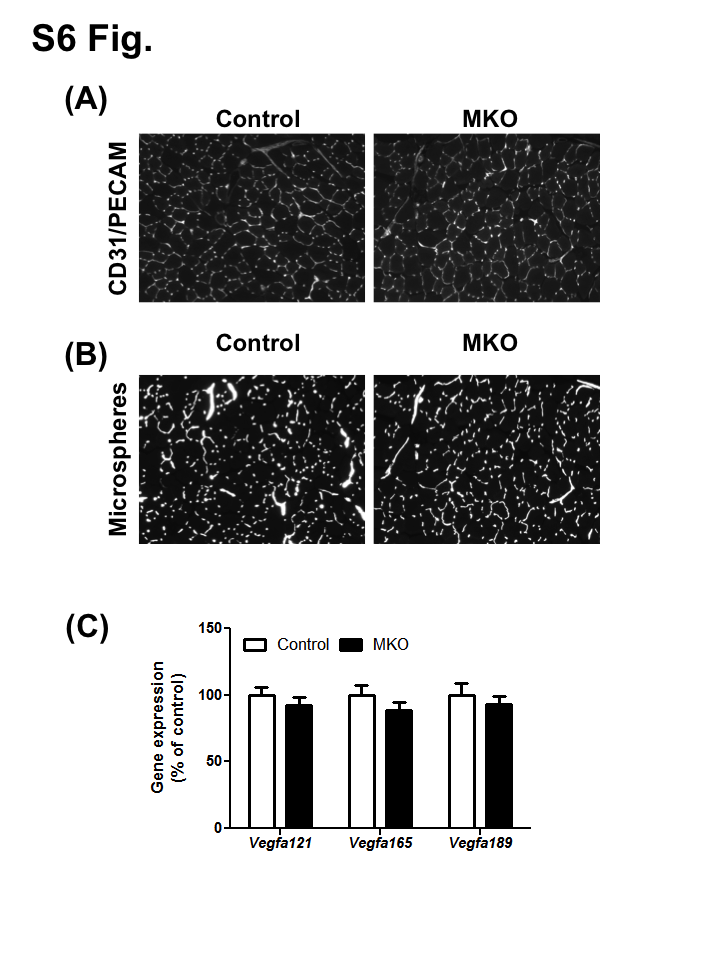

Supplement: S6 Fig — (A) Immunostaining for the endothelial marker CD31/PECAM-1 in the TA muscle cross-sections of 7 months old control and MKO mice (N = 4). (B) Representative cryo-section images of TA muscles from 7 months old control and MKO mice, perfused with fluorescent microspheres (N = 4). (C) Gene expression of Vegfa isoforms (N = 8–7). (Scale bar = 200 μm). (p = NS, Unpaired Student’s t-test.) (TIFF) [file pone.0168457.s006.tiff]

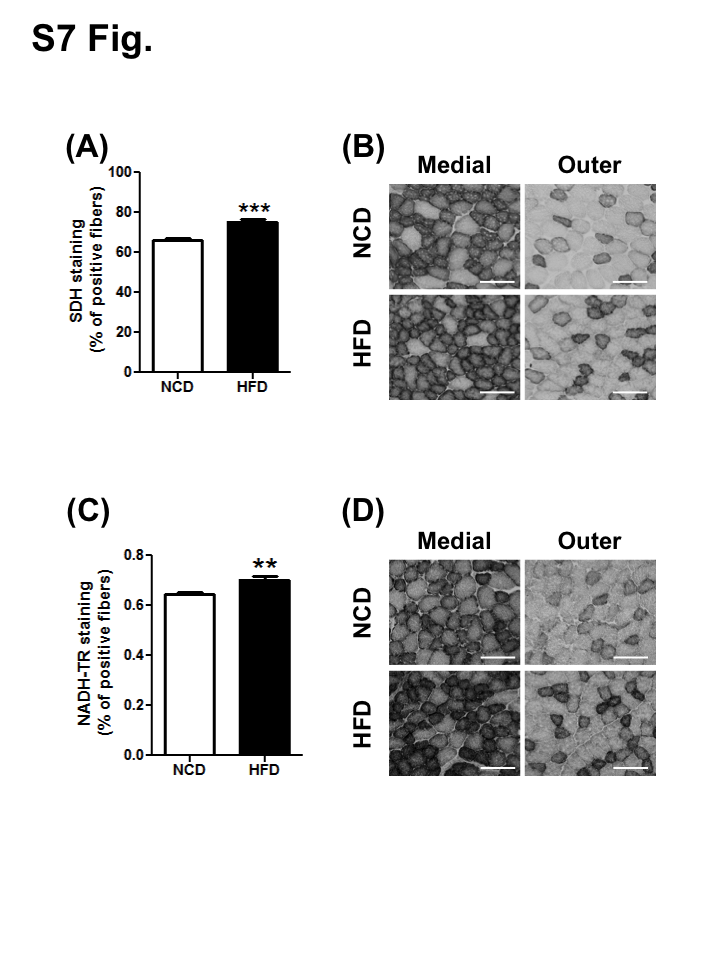

Supplement: S7 Fig — (A-B) SDH activity staining in TA muscle cross-sections from 4–5 months old NCD and HFD-fed mice (N = 5–6). (A) Representative images of the outer and medial TA muscle. (B) Percentage of SDH positive myofibers. (C-D) NADH-TR activity staining in TA muscles of 4–5 months old NCD and HFD-fed mice (N = 6). (C) Representative images of the outer and medial TA muscle. (D) Percentage of NADH-TR positive myofibers. (Scale bar = 200 μm). (**p<0.01, ***p<0.001, Unpaired Student’s t test.) (TIFF) [file pone.0168457.s007.tiff]

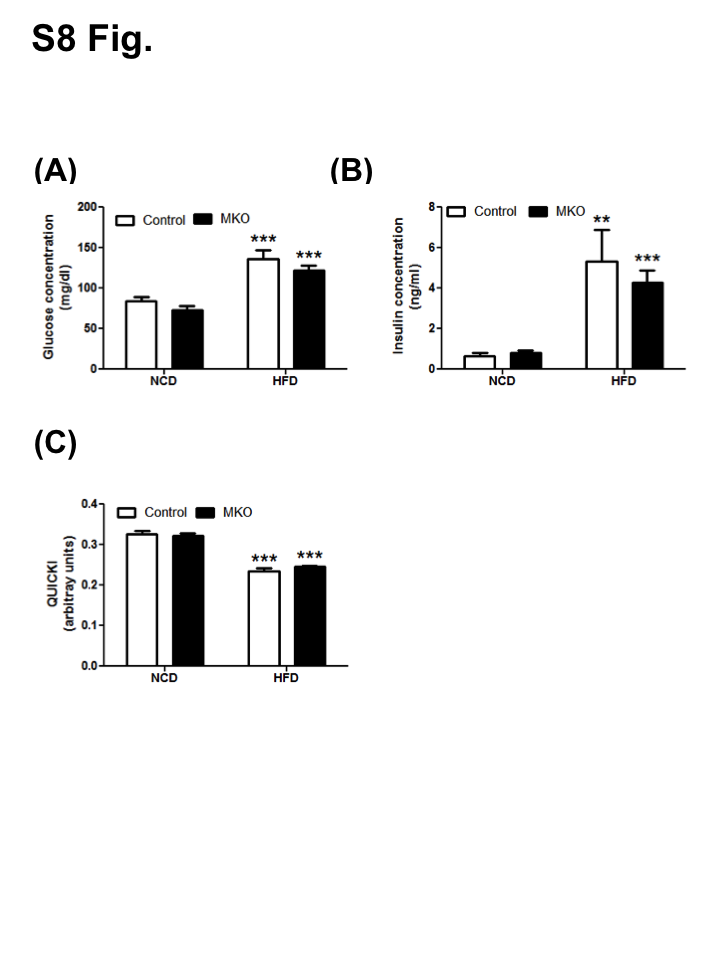

Supplement: S8 Fig — (A-B) Fasting plasma glucose (A) and insulin (B) levels in 6 hr. fasted 5 months old control and MKO NCD or HFD-fed mice (N = 5–6). (C) Quicki index in 5 months old control and MKO NCD or HFD-fed mice (N = 5–6). *Indicates a diet effect. **p<0.01 *** p<0.001 (Two-way ANOVA with a Bonferroni’s repeated measure test). (TIFF) [file pone.0168457.s008.tiff]

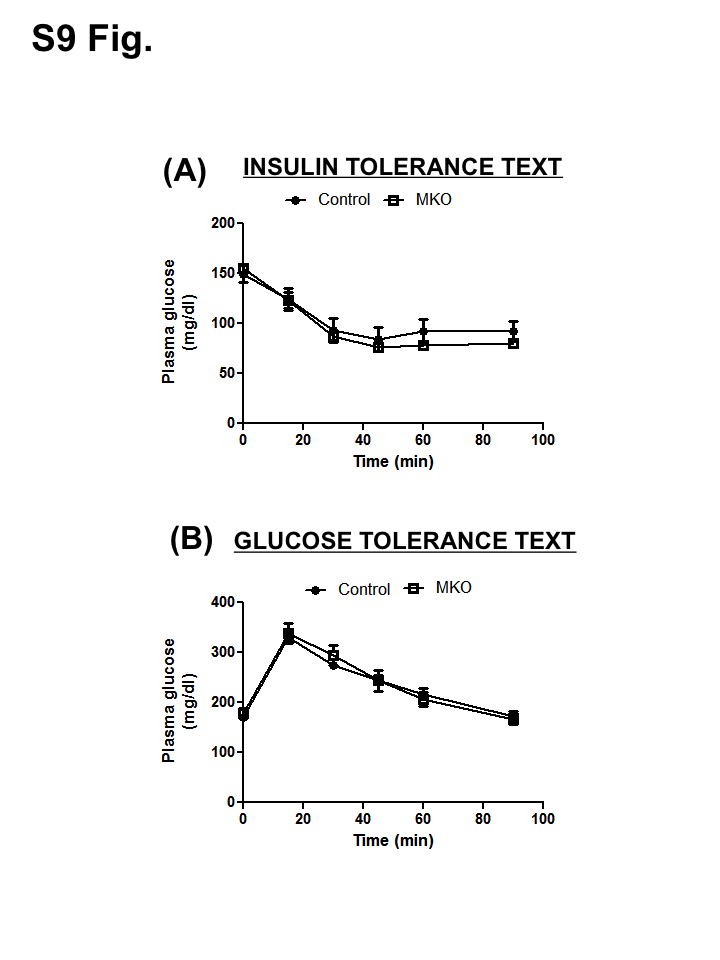

Supplement: S9 Fig — (A-B) Insulin tolerance test (A) and Glucose tolerance test (B) in 6 hr. fasted 3 months old control and MKO NCD-fed mice (N = 6–7). (p = NS, Two-way ANOVA with a Bonferroni’s repeated measure test.) (TIFF) [file pone.0168457.s009.tiff]

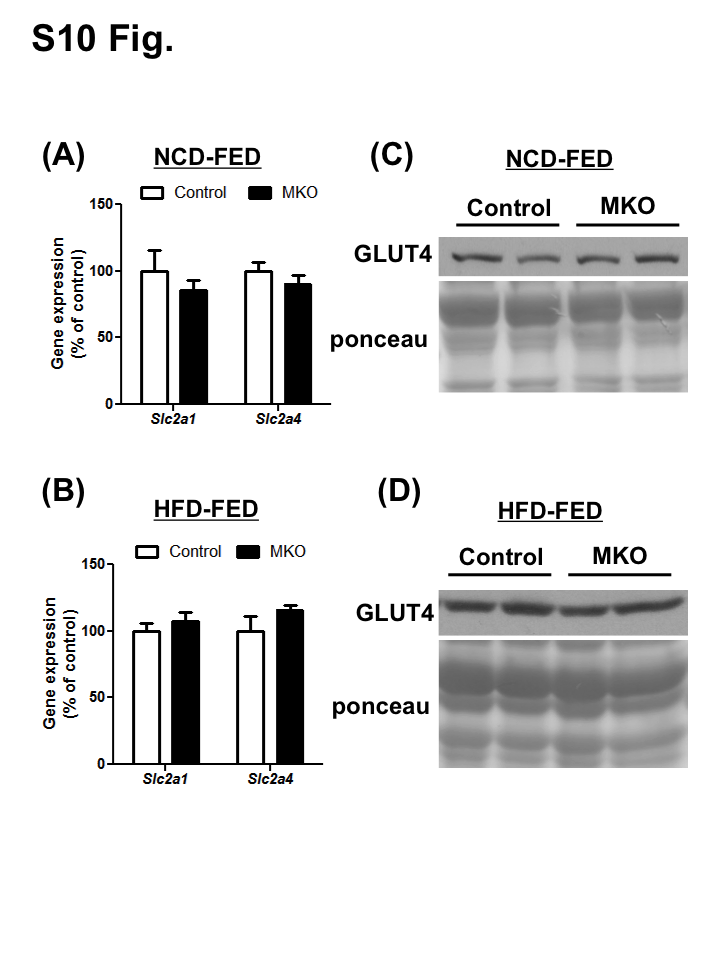

Supplement: S10 Fig — The following parameters are measured in 6 hr. fasted 5 months old control and MKO NCD or HFD-fed mice. (A) Muscle gene expression of glucose transporters (Slc2a1, Slc2a4) in NCD-fed mice (N = 4–6). (B) Muscle gene expression of glucose transporters in HFD-fed mice (N = 5–6). (C) Muscle GLUT4 protein expression in NCD-fed mice (N = 4–6). (D) Muscle GLUT4 protein expression in HFD-fed mice (N = 5). (p = NS, Unpaired Student’s t-test.) (TIFF) [file pone.0168457.s010.tiff]
