# Supplementary material for: Muscle Arnt/Hif1β Is Dispensable in Myofiber Type Determination, Vascularization and Insulin Sensitivity
Source: PLoS One. 2016 Dec 22;11(12):e0168457. doi: 10.1371/journal.pone.0168457 (PMC5178999; doi:10.1371/journal.pone.0168457)
Supplement: S1 Table — (PDF) [file pone.0168457.s011.pdf]

SUPPLEMENTAL TABLES

S1 Table. Tissue weights in 4 month-old male control and MKO mice.

	Control	MKO	p value
Quadriceps	215.7±9.4	204.8±3.5	NS
Tibialis Anterior	51.5±1.2	47.3±1.4	NS
Gastrocnemius	135±6.4	122.4±4.5	NS
Perigonadic WAT	136.8±16.59	147.8±10.94	NS
BAT	64.63±5.8	68.20±3.7	NS
