# Supplementary material for: Muscle Arnt/Hif1β Is Dispensable in Myofiber Type Determination, Vascularization and Insulin Sensitivity
Source: PLoS One. 2016 Dec 22;11(12):e0168457. doi: 10.1371/journal.pone.0168457 (PMC5178999; doi:10.1371/journal.pone.0168457)
Supplement: S2 Table — (PDF) [file pone.0168457.s012.pdf]

S2 Table. QPCR primer sequences.

	Forward	Reverse
Acadm	**5’-AGGGTTTAGTTTTGAGTTGACGG-3’**	**5’-CCCCGCTTTTGTCATATTCCG-3’**
Arnt	**5’-CATCATCGGTCTAGTTCCAGTG-3’**	**5’-CAGCATGGACAGCATTTCTTG-3’**
Cd36	**5’-CTGGGACCATTGGTGATGAAA-3’**	**5’-CACCACTCCAATCCCAAGTAAG-3’**
Cpt1b	**5’-GGGCACCTCTGGGAGTTTGT-3’**	**5’-TGGCTCACCCACACAGTGT-3’’-**
Ckmt2	**5’-ACACCCAGTGGCTATACCCTG-3’**	**5’-CCGTAGGATGCTTCATCACCC-3’**
Essra	**5’-CTCAGCTCTCTACCCAAACGC-3’**	**5’-CCGCTTGGTGATCTCACACTC-3’**
Essrg	**5’-GTTTTAAAGGCCCTTGGTGATCTCGC-3’**	**5’-CTGCAACCCTTGGACTGCCAGAAC-3’**
Hprt	**5’-GCTCGAGATGTCATGAAGGAGAT-3’**	**5’-AAAGAACTTATAGCCCCCCTTGA-3’**
Ldha	**5’-TGTCTCCAGCAAAGACTACTGT-3’**	**5’-GACTGTACTTGACAATGTTGGGA-3’**
Mb	**5’-CTGTTTAAGACTCACCCTGAGAC-3’**	**5’-GGTGCAACCATGCTTCTTCA-3’**
Myh1	**5’-CTCCAGGCTGCTTTAGAGGAA-3’**	**5’-CCTGCTCCTAATCTCAGCATCC-3’**
Myh2	**5’-AAATCTTACAAGAGACAAGCTGAGG-3’**	**5’-TGCGGAACTTGGATAGATTTG-3’**
Myh4	**5’- GAAGAGCCGAGAGGTTCACAC-3’**	**5’- CAGGACAGTGACAAAGAACGTC-3’**
Myh7	**5’-ACTGTCAACACTAAGAGGGTCA-3’**	**5’-TTGGATGATTTGATCTTCCAGGG-3’**
Pdhk4	**5’-AAGCAAAACACAAACACGAGTA-3’**	**5’CCCGGGTCATCCAACCA-3’**
Ppara	**5’-ACAAGGCCTCAGGGTACCA-3’**	**5’-GCCGAAAGAAGCCCTTACAG-3’**
Ppard	**5’-GCCTCGGGCTTCCACTAC-3’**	**5’-AGATCCGATCGCACTTCTCA-3’**
Ppargc1a	**5’-AACCACACCCACAGGATCAGA-3’**	**5’-TCTTCGCTTTATTGCTCCATGA-3’**
Ppargc1b	**5’-TCCTGTAAAAGCCCGGAGTAT-3’**	**5’-GCTCTGGTAGGGGCAGTGA-3’**
Ppia	**5’-TGGAGAGCACCAAGACAGACA-3’**	**5’-TGCCGGAGTCGACAATGAT-3’**
Slc2a1	**5’-CAGTTCGGCTATAACACTGGTG-3’**	**5’-GCCCCCGACAGACAGAGAAGATG-3’**
Slc2a4	**5’-GTGACTGGAACACTGGTCCTA-3’**	**5’-CCAGCCACGTTGCATTGTAG-3’**
Tnni1	**5’-ATGCCGGAAGTTGAGAGGAAA-3’**	**5’-TCCGAGAGGTAACGCACCTT-3’**
Vegfa	**5’-CGAGATAGAGTACATCTTCAAGCC-3’**	**5’-TCATCGTTACAGCAGCCTGC-3’**
Vegfa121	**5’-TGCAGGCTGCTGTAACGATG-3’**	**5’-CCTCGGCTTGTCACATTTTTCT-3’**
Vegfa165	**5’-TGCAGGCTGCTGTAACGATG-3’**	**5’-GAACAAGGCTCACAGTGATTTTCT-3’**
Vegfa189	**5’-TGCAGGCTGCTGTAACGATG-3’**	**5’-TCCAGGATTTAAACCGGGATT-3’**
